# The Large Scale Machine Learning in an Artificial Society: Prediction of the Ebola Outbreak in Beijing

**DOI:** 10.1155/2015/531650

**Published:** 2015-09-20

**Authors:** Peng Zhang, Bin Chen, Liang Ma, Zhen Li, Zhichao Song, Wei Duan, Xiaogang Qiu

**Affiliations:** College of Information and Management, National University of Defense Technology, Changsha 410073, China

## Abstract

Ebola virus disease (EVD) distinguishes its feature as high infectivity and mortality. Thus, it is urgent for governments to draw up emergency plans against Ebola. However, it is hard to predict the possible epidemic situations in practice. Luckily, in recent years, computational experiments based on artificial society appeared, providing a new approach to study the propagation of EVD and analyze the corresponding interventions. Therefore, the rationality of artificial society is the key to the accuracy and reliability of experiment results. Individuals' behaviors along with travel mode directly affect the propagation among individuals. Firstly, artificial Beijing is reconstructed based on geodemographics and machine learning is involved to optimize individuals' behaviors. Meanwhile, Ebola course model and propagation model are built, according to the parameters in West Africa. Subsequently, propagation mechanism of EVD is analyzed, epidemic scenario is predicted, and corresponding interventions are presented. Finally, by simulating the emergency responses of Chinese government, the conclusion is finally drawn that Ebola is impossible to outbreak in large scale in the city of Beijing.

## 1. Introduction

Ebola epidemic in West Africa has aroused high concern and begun to spread to other regions recently. EVD spreads through body fluids, with high infectivity and mortality [1]. Up to November 26, 2014, about 15935 infections along with 5689 death cases have been reported. World Health Organization (WHO) declared that all countries would pay attention to Ebola emergency and provide necessary medical aids to these countries such as Guinea, Liberia, Nigeria, and Sierra Leone [2]. Up to now, the battle against EVD is ongoing and many governments have made emergency plans. Moreover, vaccines against EVD are under test, and it will come into use [3]. Recently, the spokesman of Health Ministry has declared that Ebola would not outbreak in China in large scale, though the imported risk of EVD exists.

In China, dating back to the outbreak of Wenchuan earthquake in 2008, Natural Science Foundation of China (NSFC) has already carried out a major research plan of unconventional emergency management. Supported by NSFC, National University of Defense Technology (NUDT) has established the platform of computational experiments [4]. Meanwhile, artificial Beijing is generated according to the geodemographics data [5]. Additionally, confirmatory experiments, such as the propagation of H1N1 pandemic influenza, have proved the validation of artificial Beijing [6]. However, it has also exposed some drawbacks. Fixed behavior mode especially restricts the heterogeneity and self-adaption of individuals. For example, contacts always occur among the minority and cannot simulate the actual sense of epidemic. Artificial Beijing is a dynamic system where individuals' behaviors are continually evolving. Therefore, multiagent learning is involved to optimize the behaviors and travelling mode. It endows individuals with the abilities to adapt to the virtual city according to previous knowledge and current situation.

Since the outbreak of Ebola in West Africa, many significant works have been done to explore the propagation mechanisms and corresponding interventions. Some foreign scholars have distilled the propagation parameters of EDV using the first-hand data by investigation [7]. Simultaneously, domestic scholars have also predicted the outbreak of Ebola in China by analytic method [8]. However, they always neglect the actual interaction conditions among individuals, which always led to the amazingly increased infections and death cases. In our study, the prediction of Ebola emergency is based on artificial Beijing, where social networks, individuals' behaviors, and environment factors are considered at the same time. Subsequently, the occupations of infection cases are summarized, the infection locations are analyzed, and interventions such as isolation and immunization are also discussed. Noncontact infections especially are analyzed in our design. In addition, the infections of medical workers and the corresponding interventions are also discussed. Finally, four levels of emergency responses are simulated, according to the current emergency plans of Chinese government.

## 2. Reconstruction and Optimization of Artificial Beijing

### 2.1. Reconstruction of Artificial Beijing

**Figure 1:**
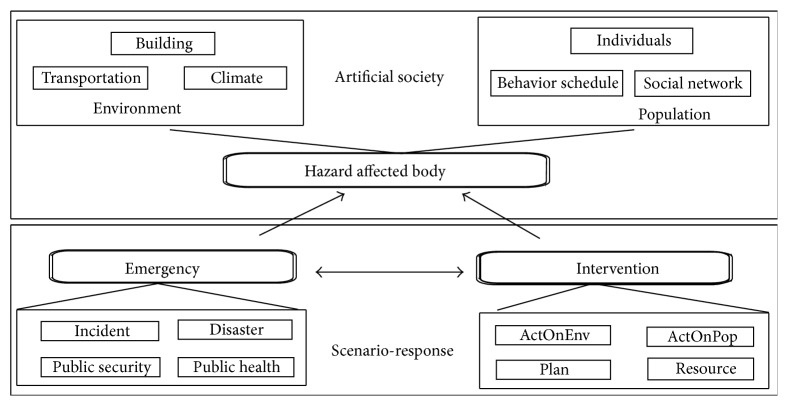
The relationship between PSTT theory and ACP approach.

Emergencies, hazard affected bodies and interventions are viewed as the core parts of the public security triangular theory (PSTT) [9]. In addition, according to ACP approach, artificial society is the foundation of computational experiments [10]. As shown in Figure 1, hazard affected bodies are just the components of artificial society, while emergencies and interventions are the cores of scenario-response theory [11]. Therefore, population is the foundation of artificial society, and environment provides the places for activities. Generally, emergencies always break the internal balance of artificial society, and then interventions will revise the states to normal.

In artificial Beijing, about 19.6 millions of individuals and 8 millions of buildings are generated. Based on census data, households are generated and individuals are endowed with social roles such as infant, student, worker, elder, and the unemployed. What is more, multiple social networks are involved including family, classmate, neighborhood, and coworker. Simultaneously, types of environment are designed such as house buildings, workplaces, educational institutions, consumption locations, entertainment locations, and medical institutions. According to the social roles, behavior schedules of individuals are designed. As shown in Table 1, basic schedules of student specify the location, duration, and probability of each activity [12]. *p*_*i*_ in the table means the action probability in the relevant period. In addition, correlative locations are assigned for each individual. For instance, the correlative locations of a student include dormitory, classroom, library, playground, and restaurant. The detailed generation process of artificial Beijing has been discussed in literature [13], contributed by another member of our team.

Subsequently, domain-oriented computational experiments are supported by artificial Beijing, such as epidemic propagation [14], rumor spreading, and traffic evacuation. In the study of Ebola epidemic, it is necessary to expand the corresponding attributes of population and environment. Therefore, typical occupations are designed to simulate the main populations in the virtual city, including medical workers, students, workers, and retirees. Additionally, individual attributes such as age, occupation, and health state are involved. Simultaneously, typical environments such as residential buildings, hospitals, and restaurants are considered. Residential buildings are viewed as the main areas of Ebola propagation because of high contact frequency among families. Hospitals are the places for treatment and isolation, while restaurants provide the places to establish the temporary group with weak links [6], where EVD spreads by noncontact infections. Additionally, the capacity and contact frequency of environment are also considered.

### 2.2. Optimization on Individuals' Behaviors Based on Machine Learning

As previously discussed, basic schedules define the daily activities of individuals. However, some problems are exposed in artificial Beijing. On the one hand, large scale individual-based simulation brings amazing overhead in computing and communication. Moreover, interventions will also directly bring extra-cost by replanning the schedules of individuals dynamically. On the other hand, it is hard to depict the activities of individuals, which is associated with the reasonability of artificial Beijing. Furthermore, it will affect the interactions among individuals, directly related to the accuracy and reliability of the prediction of Ebola epidemic. Therefore, two ways are outlined to optimize the performance: (1) improving parallel engine by introducing new technologies and algorithms and (2) optimizing the behaviors of individuals and the structure of artificial Beijing. In this section, detailed optimization on individuals' behaviors will be discussed, which not only decreases the computing consume, but also improves the reliability of the virtual city.

**Figure 2:**
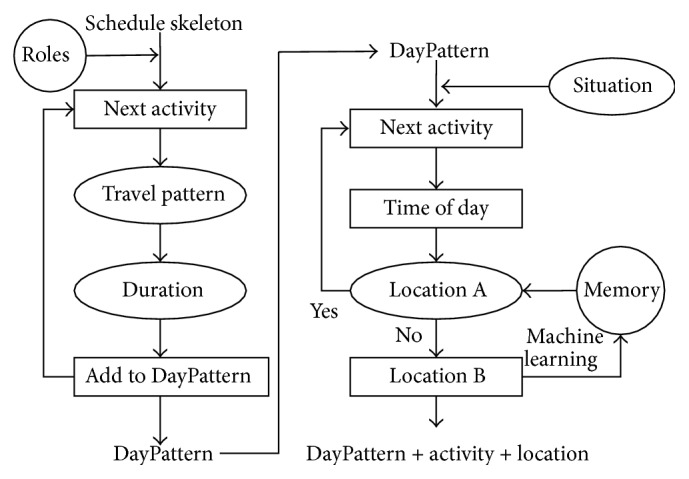
Schedule, DayPattern, and activity.

In our study, behaviors schedules are replanned every day according to the history operation and current situation. As shown in Figure 2,* DayPattern* and* activities* compose the skeleton of behaviors schedules.* DayPattern* is designed according to the social roles of individuals, which contains activity items, durations, and the travel patterns between two* activities*. For instance, the* DayPattern* of a student may consist of breakfast, study, lunch, rest, sport, dinner, entertainment, and sleep. Subsequently, detailed activities are specified according to* Daypattern* and current situation. Usually, each* activity* corresponds to a location (*l*) and duration (Δ*t*), stored in the memory (*M*) of computer. It is priority for an individual to take an activity in the memory and choose the predesigned location. However, an individual needs to replan its* activities* sometimes. Most of* activities* will especially be replanned under emergency situation. In addition, an individual just needs to choose another location for* activities* in case that the current location in the memory is unreachable.

It provides an individual with the ability to adapt to the environment and cooperate with each other. However, two kinds of difficulties are presented: the balance between exploring and choosing (an individual needs to explore information from not only environments but also other individuals) and the dimension of computing (it involves quantity discrete states and brings large consume in computing).

In our study, machine learning is introduced to solve the replanned problems, which will be discussed in detail. First of all, a location is considered feasible if the following condition is met:

(1) $$\begin{array}{c} \exists l\in G_{l},\;l\in G\left\{a\left(\tau \right)\right\}, \end{array}$$

where *τ* is an index of activities in a given schedule *S*, *G*_*l*_ is the set of facility at location *l*, and *G*{*a*(*τ*)} is the set of facilities compatible with activities of type *a*(*τ*) [15]. In our study, *l*_*N*_ is the maximum volume of *l*, and *l*_*n*_ is the current volume. If an individual enters *l* where *l*_*N*_ equals *l*_*n*_, it will search for new locations around him. In addition, Δ*t* represents the duration time of an activity, which should be in the window of the opening time (*l*_*T*_) at location *l*.

Let *L* be the choice set for the given activity defined by (1) and let *t*_*k*_ be the travel time to the location *l*(*k*) ∈ *R*_*L*_; then *R*_*L*_⊆*L* is the subset of locations reachable. If *t*_1_ < *t*_2_ < ⋯<*t*_*m*_ is the ranking of *t*_*k*_, then the heuristics of machine learning are listed as follows:

(H1): if *l*(*k*) ∈ *R*_*L*_, then rank *t*_1_ < *t*_2_ < ⋯<*t*_*m*_;
(H2): if *l*_*N*_ = *l*_*n*_, then remove *l*(*k*) from *R*_*L*_;
(H3): if Δ*t*⊈*l*_*T*_, then remove *l*(*k*) from *R*_*L*_;
(H4): if (*l*(*k*) ∈ *R*_*L*_)⋀(*l*(*k*′) ∈ *R*_*L*_∣*t*_*k*_ = min*t*_*k*′_)⋀(Δ*t*⊆*l*_*T*_), then choose *l*(*k*).

The algorithm starts from (H1). If *l*′(*k*)  is full or *l*′(*k*) is closed, then *l*′(*k*) will be removed from *R*_*L*_ and the algorithm returns to (H1). (H4) illustrates that individuals would choose the feasible location with the minimal travel time.

Let 〈*τ*, Δ*t*, *l*〉 be an activity of individual (*j*); then its behavior schedules (*S*_*j*_) are formalized as

(2) $$\begin{array}{c} S_{j}=\overset{m}{\underset{i=1}{\sum }}\langle \;\tau_{ji},{\Delta t}_{ji},l_{ji}\;\rangle , \end{array}$$

where *m* is the total of activities and ∑_*i*=1_^*m*^Δ*t*_*ji*_ = 24 h. Subsequently, if *S* represents the whole schedules of individuals, *n* is the total of individuals, and then it is formalized as follows:

(3) $$\begin{array}{c} S=\overset{n}{\underset{j}{\sum }}S_{j}=\overset{n}{\underset{j}{\sum }}\overset{m}{\underset{i=1}{\sum }}\langle \;\tau_{ji},{\Delta t}_{ji},l_{ji}\;\rangle . \end{array}$$

Finally, *S* is stored in the memory of computer, and decreases the computing consume in operation. This algorithm is suitable for the large scale individual-based system. First, it just needs to update minority individuals in each step. Actually, only minority of activities is replanned under emergency or in case that locations are unreachable. Second, it is efficient to specify the daily activities of individuals, because most activities along with the locations are restored in the memory.

After continual optimization, the operation of artificial society and the behaviors of individuals would be reasonable. In our design, behavior schedules are designed based on history operation and current situation. Optimized schedules provide individuals with more freedom to adapt to environment, and it is especially important for them to react under emergency. Moreover, dynamic schedules are able to simulate their behaviors in emergency management. For example, an individual will keep far away from the epidemic areas if epidemic outbreaks, while he may be isolated at home under interventions.

## 3. Models

The main characteristics of EVD include short incubation, high mortality, and fluid shift. In this section, the course model along with propagation model will be established.

### 3.1. Ebola Course Model

According to the epidemiology classification, individuals are divided into 4 categories: the susceptible, the exposed, the infected, and the removed [16]. As for EVD, susceptible and exposed individuals have no infectivity, while infected individuals are able to infect others. Removed individuals include death ones and discharged ones. Assuming that the total number is *N*, four categories of individuals at time *t* is *S*(*t*), *E*(*t*), *I*(*t*), and *R*(*t*), and then *S*(*t*) + *E*(*t*) + *I*(*t*) + *R*(*t*) = *N*,

(4) $$\begin{array}{c} S\overset{\alpha SI}{\to }E\overset{\beta E}{\to }I\overset{\gamma I}{\to }R. \end{array}$$

The dynamic process of disease course is shown in (4). *αSI* especially is the infection probability of SEIR model. Exposed individuals could be transferred to the infected at the rate of *β*  in a unit time, while removed rate from the infected is *γ*. Subsequently, it is easy to get the conclusion that the incubation period is  1/*β*, and the infectious period is  1/*γ*. In addition, *γ*/*β* is viewed as the reproductive number (*R*_0_) of an infectious disease. *R*_0_ is always defined as the expected number of secondary infectious cases generated by an average infectious case in an entirely susceptible population [17]. *R*_0_ could be expressed as *R*_0_ = *kbD*. Where *k* is the contact times for each infected individual in unit time, *b* is the infection probability for per contact between infected and susceptible individuals, and *D* is the mean duration of infection. To control the epidemic situation, *R* must be maintained below 1 by interventions.

Ebola propagation parameters in West Africa have been revised by Chinese Academy of Sciences, as shown in Table 2. The average durations of exposed period and infected period are 10.2 and 5 separately; therefore, *β* and *γ* are set as 0.098 and 0.2. Then the average reproductive number *R*_0_ is 2.041, which equals *γ*/*β*. Additionally, the value is coincidence to the statics data (*R*_0_ ∈ [1.4,2.26]) in West Africa.

**Figure 3:**
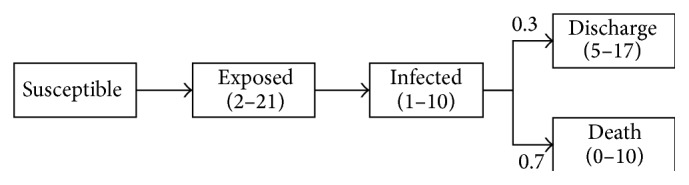
Ebola course model.

In our design, the course model of Ebola is built based on these parameters above. The exposed period lasts 2 to 21 days with no or little infectivity, while the infected period lasts 1 to 10 days with intensity infectivity. In addition, the mortality is set as 70%, according to the WHO report [8]. In the last period, the recovering time conforms to *U*(0,10) days, while the death time conforms to *U*(5,17) days. The course model and the propagation parameters are shown in Figure 3.

### 3.2. Propagation Model

As previously discussed, the main manner of propagating EVD is biologic shift. In our study, it is divided into contact propagation and noncontact propagation. Contact propagation spreads EVD by contacts between the infected and susceptible ones. In the process, contact infection probability (CIP) and contact time (CT) are discussed. According to the literature [3], EVD may survive several hours outside the bodies, and one may suffer from the virus according to polluted materials used by patients. Noncontact propagation describes the infections through polluted materials or buildings. For example, an infected individual A has ever stayed in a building and B is probably infected once entering into this building. Additionally, the noncontact infection probability (NCIP) is relevant to CIP, and it decreases along with the time (*t*) elapsing. Once no patient enters into the building, the infectivity of environment will be weakened after the duration time (*T*). In our study, exponential distribution with the weakening parameter *α* is introduced to depict the process, and *α* is predefined as 1. Assuming that CIP is *x*_1_, NCIP is *x*_2_, and their relationship is formalized as

(5) $$\begin{array}{c} x_{2}=x_{1}*e^{-\alpha \left(T-t\right)/T},\;t\in \left[0,T\right]. \end{array}$$

In our design, contact infections mainly consider the contact frequency of individuals in different types of environments, while noncontact infections mainly consider the survival time of Ebola virus in special environment. As shown in Table 3, the capacity, contact frequency, and survival time of Ebola in different environments are presented.

In addition, both CIP and NCIP are related to self-protection (SP) levels. SP describes the immunity levels of individuals, associated with the usage of antibiotics, prevention broadcast and physical condition. In a sense, these factors are also positive correlation to SP. If SP is set as *y*, the actual infection probability (AIP) will be shown as follows:

(6) xAIP1=x1∗1−y,y∈0,1,xAIP2=x2∗1−y,y∈0,1,

where *x*_AIP1_ is the actual infection probability of contact infection, while *x*_AIP2_ is the actual infection probability of noncontact infection. Of course, the values of SP are different among individuals. Especially, medical workers are designed with high SP.

## 4. Prediction and Analysis of EVD Epidemic 

The scenario of Ebola propagation is set as follows. An Ebola carrier entered into the city of Beijing, and the patient was not isolated immediately as the unobvious symptoms in incubation period. Once symptoms have been exposed for a few days, the cross infections would cause the outbreak of Ebola epidemic. The main tasks of this experiment are listed as follows: (1) the propagation among typical occupations is analyzed, and corresponding measures are also discussed; (2) the infections in typical environments along with corresponding interventions are analyzed; (3) the roles of vaccines, antibiotics, and treatments are discussed; (4) the impacts on population and environment are analyzed, and the roles of government are also discussed.

### 4.1. Prediction of EVD Epidemic

As previously discussed, the infectivity is determined by infection probability and contact frequency. Contact frequency (CF) is predefined in artificial Beijing, while the infection probability (IP) will be confirmed by the propagation parameters in West Africa. Actually, the infection probability is just the internal parameter for prediction, which is different from that in medical science. The estimated reproduction numbers (*R*_0_ ∈ [1.4,2.26]) and doubling times (Dt ∈ [15.7,30.2]) are gained, according to the literature [8]. In artificial Beijing, the infection probability will be gained in the preparation experiment according to *R*_0_ and Dt.

In the preparation experiment, the basic infection probability is verified by comparing simulation result with theoretical value. Considering the differences among occupations, four root infectious cases are supposed, including a worker, a doctor, a student, and a retiree. Subsequently, 100 days of propagation is simulated at different infection probabilities. The interaction experiment refers to millions of individuals and leads to huge consumption in communication and computation. The operation environment consists of 24 cores and 128 GB memories. It will take 1.5 hours to simulate the whole process, if simulation step is set as 10 minutes. Each sample will run 10 times and calculate the average *R*_0_. The detailed parameters are listed in Table 4.

Actually, low infection probability always results in failure of propagation, and thus it is hard to simulate the outbreak of epidemic. In contrast, high infection probability always results in high infection velocity and amazingly increased infection cases. By comparison and analysis, the infection probability is finally set as 0.01, where *R*_0_ is 2.2108 and Dt is 21. Although *R*_0_ is a little higher than the average value in West Africa, it is still reasonable to predict the epidemic spreading in Beijing. On the one hand, effective contacts are always restricted by many factors, which would lead to low infections; on the other hand, the population density of Beijing is higher than that of Africa.

**Figure 4:**
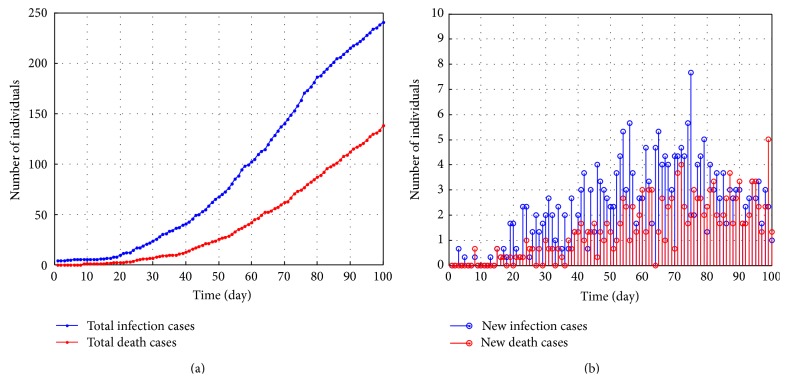
Total infection cases and new cases in 100 days under no interventions: (a) total infection cases and death cases; (b) new infection cases and death cases.

Based on current infection probability, the infection scenario in 100 days is simulated. The total infections will reach 240 in the 100th day along with 140 death cases. Meanwhile, both new infections and death cases are at the rate in single digits every day. As shown in Figure 4, the growth slows down in the 75th day. Actually, infection cases have reached saturation in the initial social networks. For instance, an infectious student has infected his families, friends, and classmates as much as possible during this period. Meanwhile, these individuals just compose a relative isolated social network. Once the infection transfers to another group, new growth will emerge.

**Figure 5:**
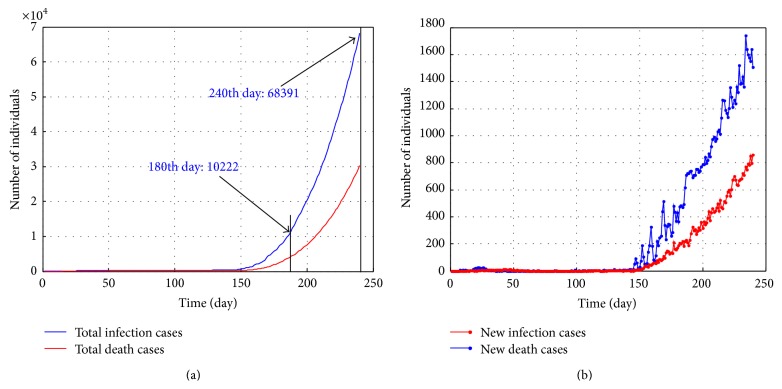
Total infection cases and new cases in 180 days and 240 days under no interventions: (a) total infection cases and death cases; (b) new infection cases and death cases.

Moreover, the situations in 180 days and 240 days are also predicted. As shown in Figure 5, the infections emerge exponentially in the 150th day. The infection cases will reach 10 thousands in the 180th day, and new cases are about one hundred per day. Subsequently, the infection cases will reach 68391 in the 240th day, and new cases are about 1 thousand per day. Of course, the result is gained in the absence of interventions and it is impossible in practice.

### 4.2. Experiment Analysis

As previously discussed, the incubation and infectious periods are 10.2 and 5 separately. Therefore, the average period of propagation is 15.2, which equals the sum of 10.2 and 5. By computing, the average generations (AG) in 100 days, 180 days, and 240 days are 6.58, 11.8, and 15.79, respectively. Additionally, the average reproductive number (*R*_0_) is 2.041. Subsequently, the infected cases at different times can be calculated. As shown in Table 5, the simulation results (SR) and the theoretical infections (TI) are basically in the same magnitude. Of course, the simulation infections are smaller than theoretical values. However, it is rational because of the rigorous interaction conditions in experiment. In a word, it predicts the possible epidemic situations in a long time, and the simulation result is reliable in a sense.

**Figure 6:**
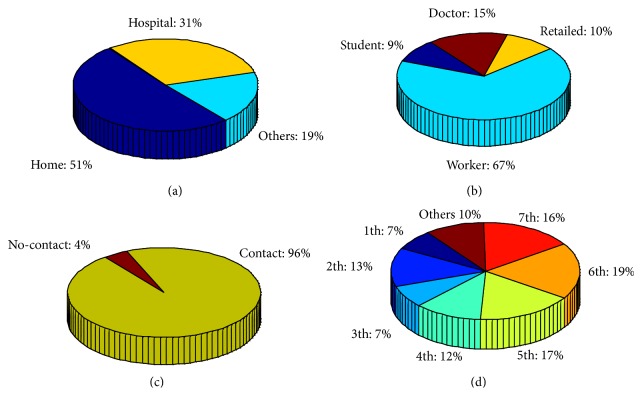
Detailed infections in 100 days under no interventions: (a) infection locations at home, hospital, and other places; (b) the occupation type of infections in worker, student, doctor, and retailed individuals; (c) the proportion between noncontact infections and contact infections; (d) the proportion of different propagation generations.

In the prediction, detailed infections of occupations, environments, and infection manners are also analyzed. As shown in Figure 6(a), residential buildings are the main places for propagation, which take the proportion of 51%. It shows that families are the most possible infections if no interventions are taken. As shown in Figure 6(b), medical workers are always at high-risk environment, and infected medical workers take the proportion of 15% although they only take a small part in the whole population. As shown in Figure 6(c), noncontact infection cases take the proportion of 4%, which mainly takes place in restaurants or hospitals. In addition, the proportions of different propagation generations are also shown in Figure 6(d).

## 5. Sensitivity Experiments

In the section, a series of sensitivity experiments are presented to analyze the validity of interventions against Ebola. Traditional interventions mainly include isolation of symptomatic cases, observation of asymptomatic individuals, and inoculation on focus groups. In our design, the ratio of seeing a doctor (RSD), the time of seeing a doctor (TSD), isolation ratio (ISR), immunization ratio (IMR), disinfection degree (DD), and self-protection (SP) are analyzed and discussed in detail. According to the experiences with H1N1 and SARS, the infection cases are at the level of hundreds. Therefore, sensitivity experiments just simulate the epidemic spreading in 100 days and then analyze the validity of interventions.

### 5.1. The Ratio of Seeing a Doctor

The ratio of seeing a doctor is able to affect the whole infections directly. Once an exposed patient contacts with other individuals, it is possible to spread EVD at the same time. In addition, the polluted materials also have infectivity since EDV is able to survive for several hours outside the host. Assuming that TSD is the 1st day, RSD are set as 0.5, 0.7, and 0.9, respectively, and the corresponding infections are analyzed.

**Figure 7:**
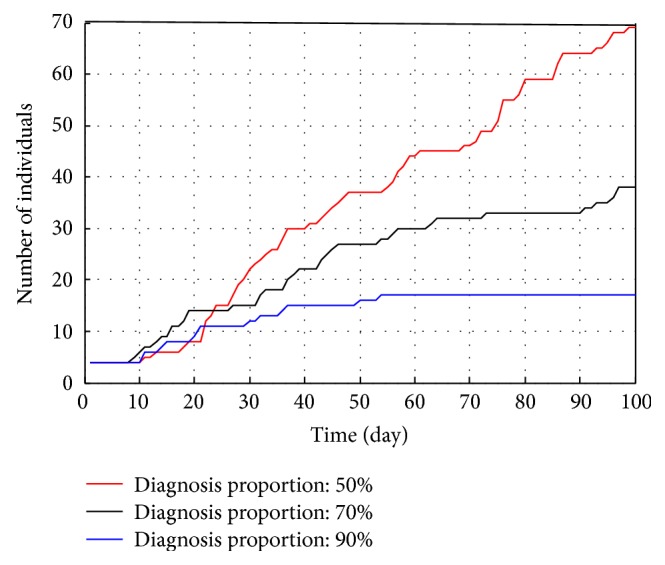
Total infections under different RSD.

As shown in Figure 7, high ratio of seeing a doctor always leads to low infection cases. If RSD reaches 0.9, the infection cases are less than 20 during 100 days, while the number will increase to 70 if the ratio is 0.5. Therefore, it is necessary to increase the RSD as much as possible.

### 5.2. The Time of Seeing a Doctor

Theoretically, early isolation and treatment will obtain better effects. In practice, EVD carriers are always diagnosed after the symptoms emerge. However, it is the exposed period that leads to the propagation between the infected and susceptible ones. Therefore, it is key to diagnosing Ebola carriers as early as possible. Experimental hypothesis is that there is only one initial carrier, and all subsequent infections will be sent to hospital. In our design, TSD is in the first day, second day and third day after the symptom exposes while RSD is set as 70%.

**Figure 8:**
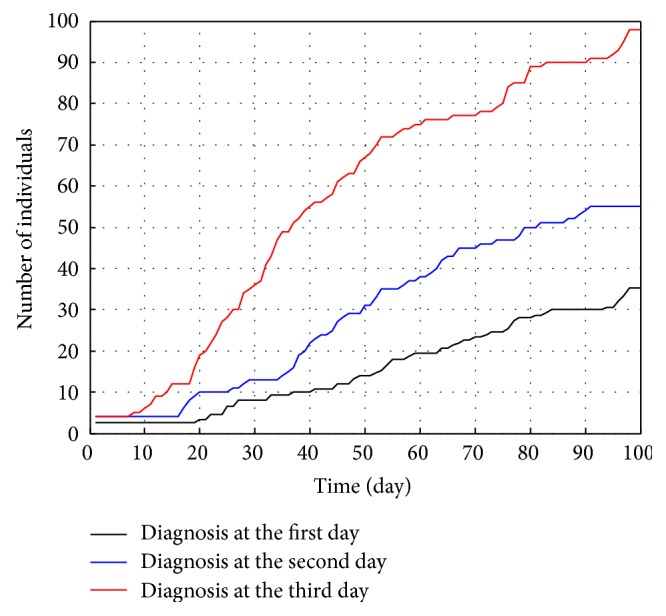
Total infection cases under different TSD.

As shown in Figure 8, the sensitivity of TSD is demonstrated. If all patients are sent to hospital in the 1st day, the number of total infection cases is about 35, the 2nd day is 55, and the 3rd day soars to 98. Then it is easy to draw the conclusion that immediate treatments are necessary under the emergency of Ebola. In practice, patients are always sent to hospital in the 2nd day because they do not pay enough attention to the symptoms originally.

### 5.3. Isolation of Potential Infected Individuals

In our study, two ways of infecting Ebola are outlined: indirect contact and direct contact. Although there are no obvious symptoms and infectivity in incubation, it is also necessary to isolate the potential infected individuals, as Ebola carriers are hard to distinguish. Once a large number of Ebola carriers transfer to the exposed, epidemic will outbreak in large scale. Therefore, it is necessary to isolate them who have contacted with diagnose patients. Simultaneously, potential infections by noncontact style would also be isolated. For instance, individuals entering the infectious environment also need to be isolated.

**Figure 9:**
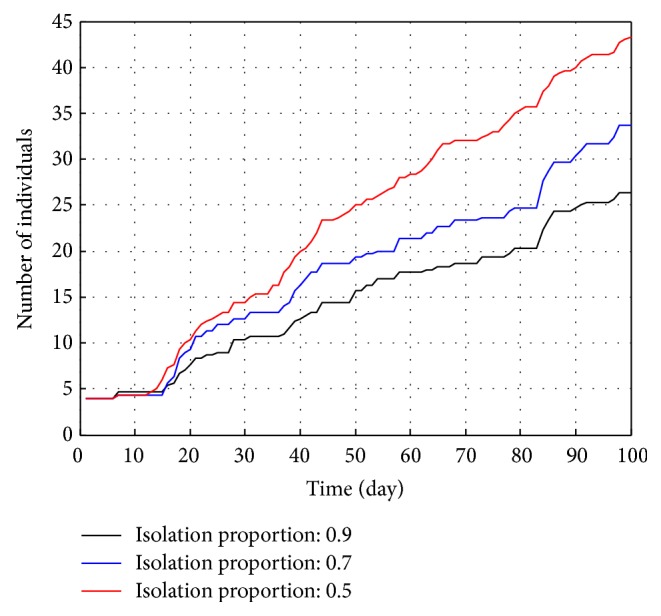
Total infections under different ISR.

Assume that TSD is the 2nd day, RSD is 0.7, and the differences are the ISR of potential infected individuals. As shown in Figure 9, ISR are set as 0.5, 0.7, and 0.9, and the epidemic situations are also analyzed separately. The result illustrates that isolating focus individuals will reduce the infection cases. The infection cases are 43 when ISR is 0.5, while the total infections have decreased to 26 if ISR is 0.9.

### 5.4. Immunization on Potential Infections

Although effective vaccines are still under test, there is no doubt that they would play an important role in the subsequent fighting against EVD. The experimental hypothesis is that effective vaccines are under manufacture and the amount is adequate. Assume that TSD is the 1st day, RSD is 0.9, ISR is 0.9, and then IMR on potential infections are discussed. As a rule of thumb, only minority of individuals may receive vaccines. Once immunization is taken, individuals will never be infected by others.

In this work, IMR are set as 10%, 20%, and 30%, and the corresponding infections are analyzed separately. As shown in Table 6, the infection cases have almost reduced half if IMR is 30%. Vaccines slow down the spreading trend obviously; however, the cost of vaccines is always expensive and the amount is always limited. Therefore, it needs to inoculate the focus groups accurately such as medical workers and potential infections.

### 5.5. Interventions on Noncontact Infections

In this part, noncontact propagation in public places is discussed. For instance, in restaurant, noncontact infections are viewed as the main manner because cross infection through dinnerware is serious. The infectivity of environment is determined by virus dose and vitality, which may be weakened by disinfection. Actually, disinfection can decrease the virus dose and reduce the virus vitality at the same time.

As shown in Table 7, noncontact infections (NCI) under different DD are analyzed. DD are set as 0.1, 0.3, 0.5, 0.7, and 0.9 separately, and the result shows that NCI decreases along with the increasing of DD. Moreover, it also reduces the total infections (TI) indirectly. Therefore, it is effective to improve DD in epidemic spreading.

### 5.6. Interventions on Medical Workers

According to the WHO report [8], medical workers (MW) account for a high proportion in the whole cases for the frequent contacts with Ebola patients. Additionally, EDV is able to survive for a period in environment due to halfway disinfection and lead to new infections in the manner of noncontact. To study the infection of MW, two propagation manners are analyzed at the same time. In the absence of interventions, SP is set as 0.2, 0.4, 0.6, and 0.8 separately, and the validity of interventions is discussed.

As shown in Table 8, the infections are associated with SP. The infected cases of MW are about 10 if SP is 0.8 while the number has increased to 30 if SP is 0.2. Moreover, these infections would infect others with different occupations and lead to the sharp increase of Ebola cases.

## 6. Four Levels of Response Strategies on Ebola Propagation 

### 6.1. Four Levels of Emergency Response Strategies

According to the emergency responses of Chinese government, there are 4 levels of response strategies against epidemic spreading. Level 4 is the weakest, including disinfection, hospital watch, and treatment; level 3 adds the isolation of familiar-contact persons and preparation of vaccines; level 2 includes trace isolation, suspending classes or works, and inoculation in small scale; level 1 is the most rigorous, in which inoculation is taken, classes are suspended, and factories are shut in large scale. In our design, different levels of response strategies are simulated, and combined interventions are estimated. As shown in Table 9, isolation, immunization, disinfection, self-protection, and diagnose factors are analyzed simultaneously. Where, LT is the time of loading interventions, TSD is the time between symptoms emerging and disease confirmed, RSD is the ratio of visiting a doctor, ISR is the isolation ratio, IMR is the immunization rate, SP is the self-protection, and DD is the disinfection degree.

**Figure 10:**
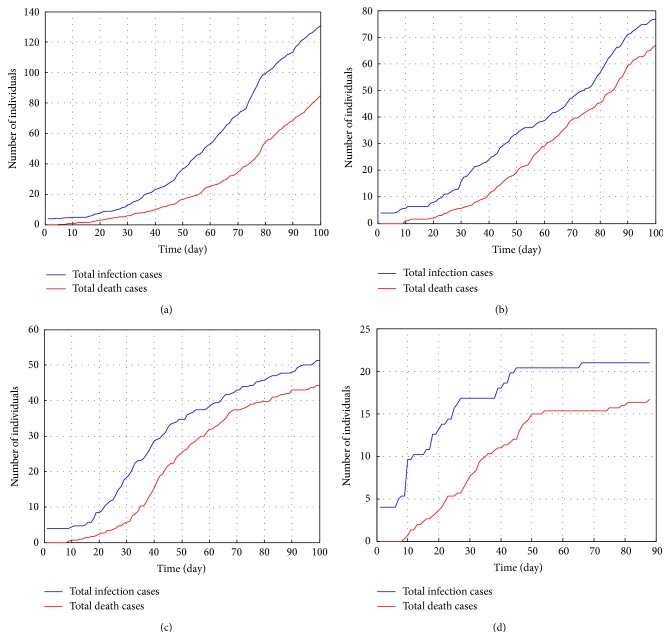
Total infection case and death case under different levels of response strategies: (a) the 4th level; (b) the 3rd level; (c) the 2nd level; (d) the 1st level.

As shown in Figure 10, epidemic situation will be under control by loading response strategies at level 2. Once taking the strongest interventions at level 1, new infection cases will decrease in a short time. Generally, rigorous interventions against Ebola will achieve better results at the cost of social orders and public resources. Luckily, since the outbreak of SARS in 2003, Chinese government has established the emergency management system, which has passed through the trail of H1N1 influenza. Although it is difficult to match all experiment parameters to the actual situation consistently, in a sense, four levels of response strategies are able to delineate the epidemic spreading under different interventions.

### 6.2. Result and Analysis

As previously discussed, response strategy at level 2 is the priority selection if a single Ebola case emerges. Under serious situation, response strategy at level 1 will be taken subsequently. The result shows that the epidemic will be under control if a single Ebola infection case emerges in the city of Beijing.

In our study, the results are credible to some degree, and the detailed analysis is listed as follows. Firstly, the construction of artificial Beijing is directly supported by NSFC. With the assistance of related institutions, the generation of individuals and buildings and their distributions meet the statistical features. Moreover, the modeling work of individuals' behaviors and social networks integrates some positive results of other research teams such as Fudan University. In addition, the reasonability of artificial Beijing has been verified in the case of H1N1 influenza, which is affirmed by NSFC. Secondly, disease model is built according to the propagation parameters in West Africa. Although these parameters may be different from that in Beijing, it has been yet revised by Chinese Academy of Sciences. Moreover, interventions are designed according to the emergency response plans, and the simulation result is coincident with the conclusions of corresponding researches. Thirdly, quantitative analysis also testifies the reasonability of simulation result. As previously discussed, *R*_0_ is the key parameter in disease propagation. Once *R*_0_ is bigger than 1, the infection disease will spread; once *R*_0_ is less than 1, the epidemic situation will be under control. In a word, the goal of interventions is to reduce *R*_0_ from the current value to below 1. In our experiments, the current *R*_0_ of 4 levels of response strategies are 1.82, 1.43, 0.93, and 0.48 separately at the 100th day. Therefore, it is reasonable to control the epidemic situation if rigorous interventions are taken immediately.

## 7. Conclusion and Discussion

### 7.1. Conclusion

The main work of this paper is summarized as follows. Firstly, artificial Beijing is reconstructed to meet the demand of epidemic spreading. In addition, individuals' behaviors are optimized by the technology of machine learning. Secondly, Ebola course model and the propagation model are also built according to the propagation parameters in West Africa. Thirdly, the epidemic situations of Ebola influenza in 100 days, 180 days, and 240 days are predicted, and the infection cases among different occupations and environments are analyzed separately.

In this paper, the propagation process of EVD and its corresponding interventions are simulated based on artificial Beijing. Moreover, diagnosis, isolation, immunization, self-protection, and disinfection are discussed. In terms of the features of fluid shift, two manners of infection (contact and noncontact) are also analyzed. Finally, it is rational to get the conclusion that it is impossible to bring the outbreak in large scale, though Ebola imported risk shall exist.

### 7.2. Discussion

This paper proposed a new method to study the disease propagation based on virtual city. The contributions of our work are outlined as follows. Firstly, artificial Beijing is generated based on demographics. Generally, some agent-based systems are always built based on simple rules, which cannot simulate the real activities of individuals. In our study, the distributions of population, environment, and social networks comply with the statistical features. Secondly, individuals' behaviors are optimized by machine learning. In our design, individuals are feasible to adjust their behaviors under special situation by replanning their schedules. Simultaneously, the optimization improves the computing performance, which involves large scale entities. Thirdly, Ebola's propagation process is simulated through the contacts among individuals. In our study, the course period and propagation characteristics of disease are two main factors in epidemic spreading. For any diseases, if we build their disease course model and propagation model, it is easy to simulate and analyze the process of epidemic spreading.

It is a scientific method with universality. With the increasing complexity of social systems, studying these problems in a traditional way becomes impossible. Although survey and qualitative analysis are able to interpret some phenomena, we can hardly explore the immanent reasons within phenomena. In our study, artificial society is a dynamic and involving virtual system, which would provide basic environment for kinds of complex experiments in social, economic, and military fields. On the one hand, it allows users to expand the attributes of existing entities to satisfy the requirement of experiments. On the other hand, we are able to cultivate the virtual society toward the desired direction and support the corresponding researches. For instance, it is necessary to cultivate the traveling mode of individuals to study personal evacuation under emergency situation. Naturally, the reliability of result mainly depends on the rationality of virtual society and the accuracy of domain models. Although solving these problems perfectly is difficult, it is still significant to build valid models and optimize the artificial society continually. In a word, this approach is universal in a sense, and it will play an especially important role in emergency management.

However, several aspects in our work still need to be improved or optimized. (1) Artificial Beijing needs to be validated constantly. Although the generation process is based on geodemographics, it is still hard to depict the actual interactions among individuals and environment. Following aspects of the virtual city also need to be improved and optimized, such as social networks, the mapping between environments and individuals, and the individuals' behaviors. Although some work has been made, it is limited to depict the behaviors perfectly. (2) Ebola parameters need to be revised constantly. Although disease models are built according to the parameters in West Africa, the differences between them cannot be ignored. Therefore, the error inevitably exists in predicting the epidemic spreading in Beijing. For instance, many cases in Africa are infected by contacting the corpse of Ebola patients, while this funeral custom is nonexistent in China. (3) Four levels of response strategies cannot match well actual situations. The motivation is just to simulate and valuate the response interventions at different degrees, and it is hard to contain all possible factors. In addition, social costs of interventions are not considered in our design. (4) Some experimental parameters are not supported by data. For instance, contact frequency and infection probability are set by the rule of thumb. Although it is significant for predicting the epidemic situation, there is really no means in medical field. In addition, it is hard to carry out sensitivity experiments in practice. Once epidemic outbreaks, interventions tend to be taken in group, but not singly.

Although some aspects need to be consummated and strengthened in our work, the predicting experiment is still significant in practice. Firstly, infections cases are gained by individuals' contacts, and it is valid to forecast and analyze the epidemic situation. Secondly, sensitivity experiments are taken to analyze the roles of key factors in interventions. Thirdly, different levels of response plans are designed, which are significant for decision makers to estimate epidemic situations and take proper actions.

## Figures and Tables

**Table 1:** Basic schedules of student in a working day.

Duration (Δ*t*)	Activity	Location	Probability
00:00–06:00	Sleep	Dormitory	*p*0 (1.00)

06:00–08:00	Breakfast	Restaurant	*p*1 (0.68)
	Sports-breakfast	Playground-restaurant	1 − *p*1 (0.32)

08:00–12:00	Class	Classroom	*p*2 (0.77)
	Study	Library	1 − *p*2 (0.23)

12:00–14:00	Lunch	Restaurant	*p*3 (0.90)
	Lunch-rest	Restaurant/dormitory	1 − *p*3 (0.10)

14:00–18:00	Class	Classroom	*p*4 (0.77)
	Study	Library	1 − *p*4 (0.23)

18:00–20:00	Dinner	Restaurant	*p*5 (0.63)
	Dinner-sports	Restaurant/playground	1 − *p*5 (0.37)

20:00–22:00	Rest	Home	*p*6 (0.63)
	Study	Classroom	1 − *p*6 (0.37)

22:00–24:00	Sleep	Home	*p*7 (1.00)

**Table 2:** Ebola propagation parameters in West Africa.

Parameter	Mean	sd	Variance
Incubation period	10.20	6.00	36.00
Infectious period	5.00	4.70	22.09
Admission to death	4.20	6.40	40.96
Admission to discharge	11.80	6.10	37.21
Generation time	15.30	9.30	86.49

**Table 3:** Detailed parameters of different buildings.

Building type	Capacity	Contact frequency	Survival time
Home	10	20	24 h
School	1000	30	12 h
Factory	1000	10	6 h
Restaurant	200	50	18 h
Hospital	1000	10	24 h

**Table 4:** The verification of infection probability.

IP	*R*_0_	Dt	Comment
0.005	1.7141	40	Failed
0.008	1.9107	27	Failed
0.01	2.2108	21	Selected
0.02	3.1105	11	Discard
0.05	4.7008	5	Discard

**Table 5:** Comparing with the theoretical and simulation infections.

Time (day)	AG	TI	SR
100	6.58	437	240
180	11.84	18647	10222
240	15.79	312330	68391

**Table 6:** Infection and death cases under different IMR.

Inoculation ratio	Infection case	Death case
10%	210	143
20%	168	98
30%	116	72

**Table 7:** Average TI and NCI under different DD.

DD	TI	NCI
0.1	230	9.8
0.3	215	8.1
0.5	197	6.9
0.7	181	5.1
0.9	168	3.2

**Table 8:** Average TI and infected MW under different SP.

SP	TI	Infected MW
0.2	216	31.05
0.4	203	24.78
0.6	184	15.22
0.8	157	10.36

**Table 9:** Detailed design of four levels of response strategies.

Response level	LT (day)	TSD (day)	RSD (%)	ISR (%)	IMR (%)	SP (%)	DD (%)
Level 4	15	4	0.5	0	0	0	0
Level 3	10	3	0.5	0.5	0	0.2	0
Level 2	7	2	0.7	0.7	0.1	0.5	0.6
Level 1	3	1	0.9	0.9	0.3	0.8	0.8
